# Exercise and High-Fat Diet in Obesity: Functional Genomics Perspectives of Two Energy Homeostasis Pillars

**DOI:** 10.3390/genes11080875

**Published:** 2020-07-31

**Authors:** Abdelaziz Ghanemi, Aicha Melouane, Mayumi Yoshioka, Jonny St-Amand

**Affiliations:** 1 Department of Molecular Medicine, Faculty of Medicine, Laval University, Québec, QC G1V 0A6, Canada; abdelaziz.Ghanemi@crchudequebec.ulaval.ca (A.G.); rimca@live.fr (A.M.); 2 Functional Genomics Laboratory, Endocrinology and Nephrology Axis, CHU de Québec-Université Laval Research Center, Québec, QC G1V 4G2, Canada; mayumi.yoshioka@crchudequebec.ulaval.ca

**Keywords:** obesity, differential genes expression, exercise, high-fat diet, pathways, potential therapeutic targets

## Abstract

The heavy impact of obesity on both the population general health and the economy makes clarifying the underlying mechanisms, identifying pharmacological targets, and developing efficient therapies for obesity of high importance. The main struggle facing obesity research is that the underlying mechanistic pathways are yet to be fully revealed. This limits both our understanding of pathogenesis and therapeutic progress toward treating the obesity epidemic. The current anti-obesity approaches are mainly a controlled diet and exercise which could have limitations. For instance, the “classical” anti-obesity approach of exercise might not be practical for patients suffering from disabilities that prevent them from routine exercise. Therefore, therapeutic alternatives are urgently required. Within this context, pharmacological agents could be relatively efficient in association to an adequate diet that remains the most efficient approach in such situation. Herein, we put a spotlight on potential therapeutic targets for obesity identified following differential genes expression-based studies aiming to find genes that are differentially expressed under diverse conditions depending on physical activity and diet (mainly high-fat), two key factors influencing obesity development and prognosis. Such functional genomics approaches contribute to elucidate the molecular mechanisms that both control obesity development and switch the genetic, biochemical, and metabolic pathways toward a specific energy balance phenotype. It is important to clarify that by “gene-related pathways”, we refer to genes, the corresponding proteins and their potential receptors, the enzymes and molecules within both the cells in the intercellular space, that are related to the activation, the regulation, or the inactivation of the gene or its corresponding protein or pathways. We believe that this emerging area of functional genomics-related exploration will not only lead to novel mechanisms but also new applications and implications along with a new generation of treatments for obesity and the related metabolic disorders especially with the modern advances in pharmacological drug targeting and functional genomics techniques.

## 1. Obesity as a Health Problem in Need of Novel Approaches

Obesity is defined as an abnormal or excessive fat accumulation [1] resulting from a broken energy homeostasis [2]. It has an epidemiological profile with a continuously increasing trend worldwide [3,4,5]. In the United States of America, at least 78.6 million people suffer from obesity [6]. Obesity is also linked to diabetes development (diabesity) [7]. In addition, not only many risk factors can increase obesity prevalence [8,9,10] but the obesity epidemic has also a major impact on health due to the complexity of its mechanisms, pathophysiology, and metabolic consequences [11]. Obesity has also been reported to increase risks and incidence of diseases and disorders such as advanced colorectal neoplasm [12], malnutrition [13], and mortality risk [14] in addition to decreasing life expectancy [15] among other diverse health impacts that could justify classifying obesity as a disease [16].

Diet control (caloric restriction), exercise, or the combination of both are the main anti-obesity approaches. For persons with morbid obesity, bariatric surgery can be an option [17] and medications are prescribed in some cases [18,19] as well. Although body weight management is a multibillion-dollar market, there are only few Food and Drug Administration-approved drugs available for long-term obesity treatment, but all have undesirable side effects [20,21].

In addition, some disabilities or heart diseases might limit the ability of individuals with obesity to exercise. In spite of the efforts of the diverse local, national, and international organizations in collaboration with health professionals and decision makers, obesity remains a major challenge with heavy consequences on life quality of the population and on healthcare budgets [22,23] especially that patients with obesity might require a specific or an adapted therapeutic care for some diseases compared to patients not suffering from obesity.

Therefore, there is an urgent need to further explore the obesity-related pathways in order to understand the underlying mechanisms and identify potential therapeutic targets. Herein, we focus on exercise and high-fat (HF) diet as they represent key factors for obesity prevention, development, and treatment area. We highlight how functional genomics allows exploring these factors via illustrative examples along with the research, pharmacological and clinical possible outcomes, and implications.

## 2. Exercise-Related Genes and Pathways: Towards an Exercise Pill

### 2.1. Exercise and Health

Along with resting energy expenditure, exercise-induced energy expenditure represents a key component of the total energy expenditure [24]. In addition to its place within the energy balance as the most variable part [24], exercise has benefits at different levels even for the older population [25]. Regular exercise contributes to reduced body weight, blood pressure, low-density lipoprotein, and total cholesterol and increases high-density lipoprotein cholesterol, muscular function, and strength as well as insulin sensitivity [26,27]. This makes exercise an important therapy both to prevent and manage obesity [28]. Although the purpose remains to create an accumulative negative caloric balance leading to weight loss [29], intensity, regularity, and duration of an exercise defines its type and the related outcomes and benefits.

The choice of exercise types depends on what we want to achieve in terms of muscle strength, fat mass loss, mitochondrial function enhancement, etc., as well as the ability of the individual depending on factors like age, cardiovascular health, and disability. For instance, an elderly person with cardiovascular disease would go for a walk to burn calories because of their limited exercise capacity [30]. The key metabolic tissue used during exercise is the skeletal muscle and its health represents a key factor for both an improved metabolic performance as well as a healthy ageing [31] which are two risk factors of obesity.

Exercise has a crucial role in maintaining skeletal muscle homeostasis [32] especially for the older population [33]. Biochemical profile of muscles is highly determined by protein synthesis (muscle contraction) and energy metabolism (energy expenditure) that govern the ability of energy usage via locomotion, which is a principle component of anti-obesity therapy involving exercise. Importantly, both body size and body composition, which are shaped by exercise, are determinants of resting energy expenditure. This shows that the benefits of exercise in terms of caloric use goes beyond the exercise-related energy expenditure. In addition, the benefits of exercise are not limited to energy metabolism, lipoprotein profile, or obesity treatment. Indeed, studies have shown how exercise could help to improve the prognosis, therapy, or prevent (reduce the risk) the onset of diverse diseases and conditions such as cancers [34,35], cancer-induced cardiac cachexia [36], multiple sclerosis [37], stroke [38], breast cancer-related lymphedema [39], as well as to counteract some treatments side effects [40] and can even be prescribed as a complimentary therapy (e.g., exercise oncology) [41].

### 2.2. Exercise Impacts Gene Expression

Identifying genes that are regulated by exercise (exercise-induced genes, especially in the skeletal muscle) has been among the focus of different research groups that have already identified a number of key exercise-related transcriptomes. For instance, numerous studies have obtained data that defined the effects of exercise on genes that are related to exercise benefits at the biochemical and metabolic levels. Indeed, they have shown that exercise induces the expression of genes that regulate or are related to mitochondrial biogenesis [42], oxidative phosphorylation (OXPHOS) [43], antioxidant defense mechanism [44], cell proliferation [45], and the amelioration of insulin resistance [46] which indicates links between exercise outcomes and transcriptome modifications.

Furthermore, other gene expression-based studies, mainly comparative [47] and under different conditions including exercise [48] and resting [49] have allowed the collation of data and increase our understanding of the skeletal muscle transcriptome and functions in diverse contexts and depending on the population category. This contributes to a more precise mechanistic understanding of the genetic and biochemical changes at the molecular level. Thus, could guide to a muscle-targeting therapy development for obesity by defining the pathway associations with genes to optimize other therapies and even improve the pharmacovigilance based on genetic profiling. Beyond that, identifying exercise-induced genes would support further progress in understanding and treating different diseases other than those only depending on energy homeostasis which would expend the benefits of “exercise pills”.

### 2.3. Gene Expression Patterns Underlie Muscular Adaptation to Exercise

Exploring such exercise-induced genes and pathways contributes to understand the molecular profiles that govern the adaptive responses of muscles to exercise. In addition, advances in epigenetics of muscle [50] in relation to exercise [51,52], diet [52], and aging [53] would further strengthen this field beyond genomics and put each of these pillars within a complementary network of data via which we can investigate potential therapies. For instance, exercise during pregnancy induces offspring changes [54,55], indicating that mother physical activity (intensity and frequency) impacts the health of the unborn child which opens an area in molecular pediatrics research.

Our team has also focused on gene expression in the skeletal muscle of endurance athletes compared to sedentary men and identified 33 genes that are differentially expressed [56]. This study, which supports the data reported above, highlight the global muscle gene expression including genes mostly related to muscle contraction and energy metabolism (two parameters improved by exercise). Moreover, these data further support our previous characterization of the global gene expression profile of sprinter’s muscle, that shows transcripts mainly involved in contraction and energy metabolism as the most expressed in muscles of sprinters [57]. Such genetic expression pattern reflects a functional and metabolic adaptation of athletes toward an increased muscle contractile function along with an enhanced energy expenditure in the context of exercise training-induced muscle adaptations [58]. Furthermore, another study, involving healthy men, shows that moderate-intensity exercise at the lactate threshold induces the expression of transcriptomes involved in the tricarboxylic acid cycle, β-oxidation, antioxidant enzymes, contractile apparatus, and electron transport in the skeletal muscle [59].

Following the same line of thought, it was demonstrated that after 6 weeks of endurance training at lactate threshold intensity, the regulation of skeletal muscle transcriptome in elderly men includes increased expression of genes related to oxidative OXPHOS [60]. All these changes reflect an increase in the energy expenditure ability via an enhanced mitochondrial activity with an increased usage of biofuels which would be combined to reduced energy storage and lead to protection from obesity. This study [60] has also highlighted the importance of mitochondrial OXPHOS and extracellular matrix (ECM) remodeling in the skeletal muscle adaptation which correlates with a previously reported work in which genes of both ECM and calcium binding are upregulated and those related to diabetes are modulated in human skeletal muscle following a 6 wks aerobic training [61]. We note that the exercise-induced genes are associated with a profile that counteracts the ageing process. Indeed, whereas ageing (risk factor for obesity) decreases metabolic performance (e.g., mitochondrial dysfunction [62]) and the strength of the muscle [63] and increases oxidative stress [64], exercise improves those biological patterns in the muscle.

One of the mild endurance training induced genes that draws particular attention is the secreted protein acidic and rich in cysteine (*SPARC*). This gene was characterized as an exercise-induced gene [60] as well as electrical pulse stimulation (considered as the in vitro form of exercise)-induced gene in C2C12 myoblasts [65]. In addition, studies have shown that SPARC increased in the skeletal muscle during training [66,67,68]. This same protein plays diverse roles in energy metabolism especially in the muscle [69,70], ECM remodeling and myoblast differentiation [71,72,73,74], inflammation [75], and cancer development [76], which would indicate that SPARC plays a role in exercise-induced benefit related processes involving inflammation, cancer, and tissue remodeling.

All these gene expression changes help to understand, at least in part, exercise-induced pathways of mitochondrial biogenesis [77] and mitochondrial biochemistry [78] as well as muscle adaptation [79] and how exercise can reverse ageing impacts on skeletal muscle [80]. Such genomics studies are supported and complemented by proteomics studies that have explored the variations in protein expression in muscle depending on the physical activity [66,81,82,83] and reflects an adaptation of the proteinic profile, comparable to the transcriptomic changes, as well. This includes the increase in the expression of a peroxisome proliferator-activated receptor γ coactivator 1 α isoform PGC-1α4 that is involved in the regulation of skeletal muscle hypertrophy [84] which reflects an aspect from the correlation and complementarity between the functional genomics and functional proteomics.

Moreover, studies of exercise-related genes can be categorized depending on exercise type, e.g., endurance-based exercise and resistance-based exercise [85]. The transcriptomic signature of exercised muscle is also variable depending on muscle fibers and age [86]. This indicates a need of a classification strategy depending of the variables (age, muscle fibers, exercise type, etc.) that modify gene expression response to exercise. Such classification could also be extrapolated to the therapeutic target identification depending on the suitable pharmacological effects (enhance the metabolism, increase muscle strength, etc.).

### 2.4. Implications

**Figure 1:**
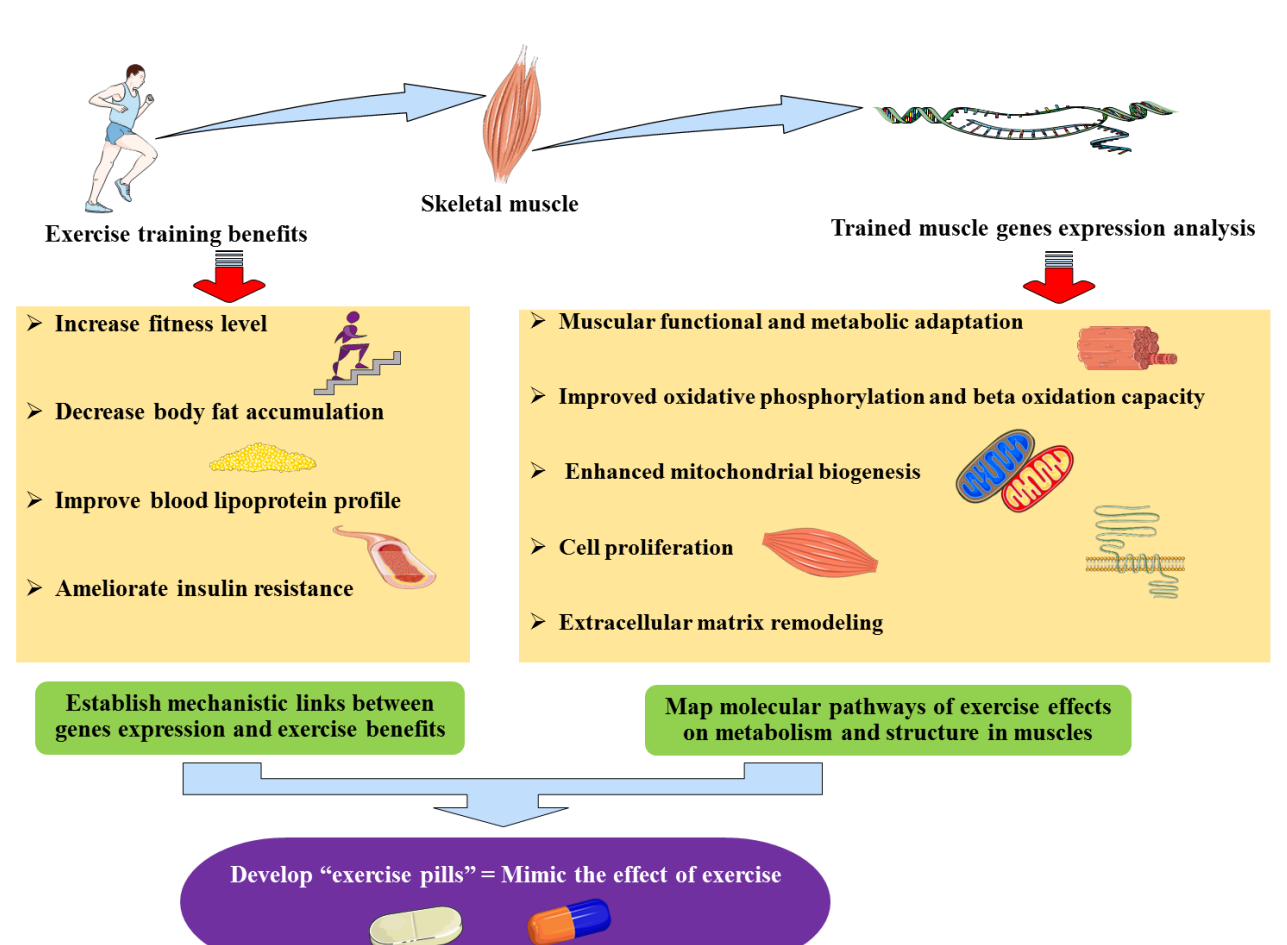
The implications of identifying genes differentially expressed during exercise training: exercise-induced genes.

Such exercise-related gene expression patterns explain some of the exercise benefits, including those seen even after detraining [87], including increased muscle contraction and energy metabolism improvement, thereby providing molecular and mechanistic links between the exercise benefits and the genes (over) expressed with or following exercise which could potentially be used for drug development towards an “exercise pill” (Figure 1).

Importantly, the exercise benefits and their clinical outcomes are precisely what clinicians hope to observe in their patients (with obesity, diabetes, etc.) such as an improved blood lipoprotein profile [88,89], increased usage of lipids and glucose, ameliorated insulin resistance, as well as an enhanced energy expenditure. Obtaining these effects is exactly what functional genomics-based therapies aim to achieve via pharmacological agents. Indeed, identifying exercise-specific genes and exploring the pathways they control would allow the development of exercise pills. Such pills could therapeutically mimic the effects of exercise via targeting these “exercise-genes” pathways through pharmacological agents and thus, obtain the benefits of exercise without intensive training. This is of a particular importance for old (and suffering from heart diseases) or disabled individuals who have limited ability to exercise but who therapeutically require the benefits of exercise. Therefore, such “exercise pill” would allow to overcome this limitation of applying exercise as a therapy for obesity.

## 3. Diet-Related Genes: A Focus on High-Fat Diet to Identify a Lipid-Specific Signal

### 3.1. High-Fat Diet Particularities in Obesity Context

As diet is the other pillar in obesity research and represents the energy intake and a key part of anti-obesity therapy, it is also an important factor for gene expression studies in the context of obesity. The diverse properties and impacts the diet has on metabolism pattern and biochemical adaptations made the identification and the exploration of associated specific gene expression patterns an important element in obesity molecular research. The effect of diet on obesity development is well known especially for HF diet [90,91,92]. The reason behind the focus on fat, beyond the concept of excess caloric intake, is that this nutrient, compared to both carbohydrates and proteins, has limited effect on satiety, is associated with high palatability, and has a high caloric density [93]. In addition, the lipid content in the modern Western diet increases fat consumption and is part of the unhealthy lifestyle. Indeed, following a HF meal ingestion, both caloric intake and energy expenditure favor weight gain because of the palatability, high caloric density, and low satiety effect of HF nutrients, as well as the weak potency for fat oxidation and energy expenditure associated with elevated fat intake [94,95,96]. The other pattern associated with HF diet is that the offspring have obesity risk and gene expression alterations [97] as a consequence of the maternal HF diet. This highlights the need to focus on HF diet especially as it impacts gene expression and epigenetics profile [98] as exemplified by studies showing that epigenetic changes can be consequences of the maternal HF diet [99,100,101]

The control of food intake represents a major determinant in the etiology of obesity especially with HF meals which acutely disrupt energy balance [102,103]. Feeding behavior is controlled by short-term circulating nutrients and hormones as well as signals derived from peripheral tissues in response to a meal and changes in energy stores. Within this context, the hypothalamus is a key brain center upon which all these peripheral signals converge to regulate feeding behavior and energy intake, thus it controls short-term as well as long-term energy balance and steady-state body weight [104,105]. Therefore, screening the changes in gene level following acute HF meal ingestions would reveal new elements within the gut–brain axis leading to the development of novel approaches for the understanding and the control of energy homeostasis. In particular, the identification of transcriptomic changes induced by HF diet both in digestive and peripheral tissues as well as within the central energy metabolism control centers in the brain.

### 3.2. Digestive System (First Food “Receptors”)

Differentially expressed genes in the stomach and intestine are key elements since these two tissues represent the sites of most of the digestive processes and where the nutrients are first available in the simplest forms (that interact with endocrine system and different receptors). Thus, stomach and intestine represent the starting point of signals controlling energy balance (including food intake). Importantly, variations (gene expression) within the digestive system may reflect changes at the digestive process that could impact the availability, the absorbance ratio, as well as the biochemical and endocrine effects of the diet nutrients. Since HF diet-induced transcriptomes would require more attention than the low-fat (LF) induced genes, it is of a great importance to identify and more precisely distinguish between HF and LF specific genes. Therefore, the particularity of selected studies we report first herein is that fasting status was the reference (control) to study both HF and LF-specific genes. In fact, numerous previous studies that investigated HF-specific changes used LF conditions as a reference, therefore, were not able to characterize LF-specific genes nor to distinguish HF-specific from LF-specific transcriptomes. We first report a transcriptomic study that identified the peripheral signals of appetite and satiety from mice duodenum by investigating the transcriptomic changes in the duodenum mucosa 30 min, 1 h, and 3 h (to explore acute impact rather than chronic gene expression modifications) following HF and LF meal ingestion [106]. This study reveals that energy, protein, and fat intake transcriptome expression changes were higher in the HF groups compared to LF groups [106,107]. These data correlate with an intestinal mucosal mRNA analysis that demonstrates changes in the expression of genes related to anabolic and catabolic lipid metabolism pathways [108] and a recent paper shows that the expression of genes related to the uptake and transport of lipid and cholesterol as well as glucose storage are upregulated in the duodenum [109]. This changes specific patterns of HF-diet compared to LF-diet. Digestive mucosa is the first tissue that interacts with nutrients during the first digestive processes and has the ability to produce signal molecules that can act as hormones within the gut–brain axis [110]. Therefore, the key concept beyond identifying digestive mucosal diet-induced genes is to eventually identify new signals and responses to nutrient ingestion controlling food intake and energy expenditure. As an example of a potential signal molecule, the trefoil factor 2 (*Tff2*) has been identified as a newly found HF-specific gene [106] for which its deficiency in mice leads to a protection from HF diet-induced obesity [111,112]. Among the hundreds of genes that are modulated after HF or LF meal ingestion [106,113,114,115,116], we put a spotlight on the *Tff2* and its pathway as a potential targetable pathway for obesity molecular therapies. Indeed, this gene is upregulated by HF (and not LF) diet [106] which suggests it is a specific acute HF-induced signals that may impact food intake regulation. At the peripheral level, HF-diet decreases the expression of genes involved in metabolizing glucose in porcine perirenal and subcutaneous adipose tissues [116] which would indicate the switch (as an adaptation) of the metabolism toward less glucose usage in the presence of lipid intake, probably to increase lipid metabolism following a LF-diet intake. In addition, it has been shown that in mesenteric adipose tissue, only LF meal upregulated transcripts implicated in lipid biosynthesis, whereas transcripts involved in lipid utilization and glucose production were downregulated in both HF and LF meals following 3 h of meal ingestion [114], also pointing a metabolic adaptation of lipid metabolism depending of lipid ratio within the diet.

### 3.3. Adipose Tissue (Energy-Stocking Tissue) and Skeletal Muscle (Energy-Usage Tissue)

HF diet induces an increase in the expression of genes related to inflammation, whereas it downregulates genes related to lipid metabolism, adipocyte differentiation markers, and detoxification processes, and cytoskeletal structural components in mouse adipose tissue [117]. These observations highlight how the metabolic function reacts to HF diet in terms of adaptation and at the same time emphasizes health problems associated with obesity such as inflammation. These results, further indicate that the metabolism is shifted toward the usage of lipids rather than glucose, are in agreement with other studies showing that HF diet enhances the expression of genes related to lipid catabolism in the skeletal muscle [118]. Such data illustrate how the metabolic cellular system can adapt to the type and the quantity of nutrients received through different diets and the activated metabolic processes are chosen depending on such factors. Exploring such “diet-oriented” metabolic pathways might allow the development of pharmacological approaches that could mimic such pathways in order to increase lipid store usage by tissues as a part of anti-obesity therapies. Importantly, knowing the metabolism-related genes regulated by diet could optimize pharmacotherapies and diet-based therapies by selecting the type and the quantity of specific nutrients that could act towards a suitable metabolic phenotype for a specific patient. Herein, it is worth emphasizing that in order to correctly design a study, selecting the control group remains critical. Indeed, to study HF or LF diet, it is important to define the reference whether it is fasting status or fed control. In case of fed control, not only the caloric content but also the fat type and its chemical nature are also to be taken into account when reaching conclusions.

### 3.4. Brain (Energy Balance-Control Centers)

Besides identifying diet-related peripheral signals, changes induced by the diet at the central level have also been studied. For instance, the study of HF and LF meal ingestion-induced changes in the hypothalamic transcriptome reveals that 3 h after the beginning of meal ingestion, 12 transcripts were regulated by food intake including two involved in mitochondrial functions [115]. This work also reveals the increased expression of the major urinary protein 1 (*Mup1*) gene in the hypothalamus of LF fed mice compared to fasting mice. MUP1 is a protein involved in metabolic profile improvement including energy balance toward skeletal muscle with increased mitochondrial function and energy expenditure in diabetic mice [119]. These MUP1 effects on metabolism regulation [120] including glucose and lipid metabolism [121], might explain the benefits of the LF diet. Such benefits are not only explained by the limited caloric intake in LF diet compared to HF diet but results from the switch of the metabolic profile toward more fuel usage and energy expenditure. In addition, we might also suggest that *Mup1,* with biochemical effects protecting from obesity, is involved in the pathways that are blunted during obesity which would further increase energy storage and decrease energy expenditure. Indeed, in another study, a 8–12 d dietary restriction in LF-diet groups of mice led to a downregulation of *Mup1* in adipose tissue [122] which could be an adaptation to the dietary restriction in order to conserve energy stores and limit energy usage since the organism is under caloric privation. This further highlights the importance of *Mup1* in energy balance, both in energy expenditure and energy conservation, and presents its function as a potential molecular target for obesity as well.

Furthermore, regarding the hypothalamic (center of energy homeostasis control) transcriptome, high-fructose diet fed to Wistar rats throughout development lead to the remodeling of 966 genes and enhanced both depressive-like and anxiety-like behaviors [123] which could lead individuals to manifest either increase or loss of their appetite. In addition, the hypothalamic transcriptome pattern under HF diet condition (over 2 wks) exploring the neuropeptides involved in energy balance explains how ingesting a HF meal contributes to remodeling the expression of neuropeptide Y, agouti-related protein, and proopiomelanocortin over time [124]. This last element is extremely important to understand the establishment and the development of obesity by studying key molecular signals at different steps and reveal the underlying paths. Importantly, the data generated on preferentially expressed genes in the hypothalamus and pituitary gland [125,126] improve the understanding of the central control of energy metabolism and diet impact on gene expression.

### 3.5. Potential Applications

**Figure 2:**
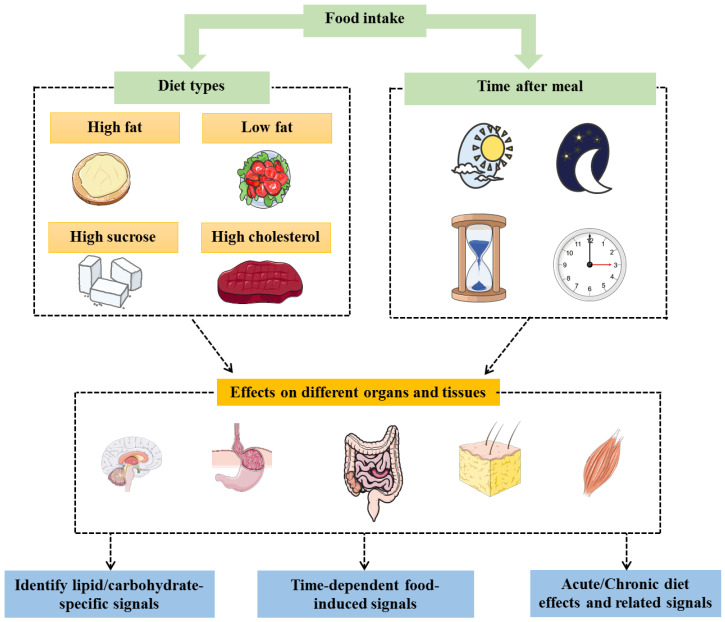
Studying the effects (expressed genes and the associated pathways) of different types of diets on the different organs/tissues involved in energy balance at different times allow to identify time-dependent specific signals (such as lipid-specific signals) regulating metabolism homeostasis.

The characterization of novel fat-specific genes may contribute to the development of new therapeutic targets for appetite and satiety controls. Herein, it is worth mentioning that the existence of two levels of diet-dependent energy metabolism control (peripheral and central) provides wider therapeutic options and further choices depending on the patient’s physiological or pathophysiological status. For instance, a patient with obesity suffering from a functional gastrointestinal disease might not respond well for an obesity therapy targeting the peripheral signals and would require targeting the central pathways. Mapping how the metabolic profiles (governed by selected genes) change according to the type of diet and the time between meal ingestion and gene expression analysis (and eventually at which time the meal is ingested) would allow the identification of selected signals that are specific and/or time dependent (Figure 2). Such data could allow to improve precise personal therapies for individuals.

Additional studies have examined the interaction between diet and gene expression regulation. HF and high-cholesterol (HFHC) diet, and HFHC plus high-sucrose diet [127] have been explored within the context of differentially expressed genes. Unlike the previous examples, blood RNA analysis was performed and revealed differential hyperlipidemia gene expression profiles even though levels of fasting plasma lipids and glucose corresponding to these two diets was similar [127]. This indicates that gene expression might not reflect phenotypic changes and that corresponding in vivo metabolic and biochemical exploration is required to understand gene expression modifications. In addition to studying the effects of diet itself, it is highly relevant to explore the impacts of drugs that modify the effects and distribution of nutrients in vivo. For example, Salomäki et al. (2014), showed that administering metformin (prescribed to regulate glucose blood levels [128]) to pregnant female mice that were on a HF diet resulted in transcriptome related to mitochondrial ATP production and adipocytes differentiation of the offspring [129] resulting in an improved metabolic phenotype. From a therapeutic viewpoint (pharmacology and nutrition), understanding the pathways stimulated or deactivated depending of the type of diets would allow nutritionists and clinicians to adapt the diet for their patients based on the therapy they are following or based on their lifestyle to avoid possible adverse interactions between the diets, therapies, and activated pathways (genes, enzymes, etc.). This would help mitigate therapeutic failure, or pharmacotoxicity by reducing the drug clearance (metabolism) that could lead to a toxic accumulation. The goal herein remains to reach and adapt to the clinical and therapeutic needs.

Finally, the main potential application beyond focusing on HF-diet-induced genes remains the fact that lipid metabolism-related feedback hormones (mainly leptin) do not have an acute effect. In fact, their effects develop after a relatively long period of time compared to carbohydrate-induced hormones (for instance insulin) that are stimulated immediately following a carbohydrate intake. This highlights the importance of elucidating changes that are both acute and specific to HF diet intake in order to identify acute signals of lipid intake; based on which therapies (hormonal or pharmacological) can be developed. In addition, HF diet changed the expression of genes related to neurogenesis, calcium signaling, and synapse, in the brain cortex [130]. Such ability of the diet to impact neuronal-specific gene patterns could explain how diet and obesity establishment affect the ability of the brain to control energy balance and would require comparable studies in the hypothalamic region, the center of metabolic homeostasis control. Combining the study of changes in the intestinal mucosa (first tissue that comes in contact with the food) with those in the brain (centers that receive peripheral signals and control food intake) would provide the best combination to identify acute HF-specific signals of food intake regulation and, therefore, optimize the therapies based on these axes.

## 4. Conclusions, Discussion, and Perspectives

Overall, identifying such differentially expressed genes related to exercise and high-fat diet and their related pathways could suggest potential novel therapeutic targets for obesity treatments after elucidating the mechanisms linking those genes to the diverse energy metabolism phenotypes. Functional genomics would, therefore, lead to a new generation of therapeutic approaches that would, through targeting selected energy balance pathways, mimic the benefits and outcomes of physical activity, suitable diets, or even hormones.

For the diet, due to the properties of lipids (high caloric density, low satiety effect, etc.), we believe that one of the best strategies to develop pharmacotherapies for obesity would be to target HF intake at the appetizer time. Therefore, one of the primary strategies is to identify and study the HF diet-induced satiety hormone; usually transcriptionally regulated 30 min to 3 h after HF meal and to deliver it at the time of appetizer in order to control HF intake, obesity, and the related complex diseases and conditions. Herein, it is important to emphasize that adequate diet control is the key solution for obesity (especially if combined with exercise [131,132]) and that pharmacological options remain complementary in selected cases. Regarding identifying pathways of the exercise-induced genes is important for development of exercise pills (long-term objective) that could therapeutically mimic the effects of exercise via targeting these “exercise-genes” pathways through pharmacological agents and, thus, obtain the benefits of exercise without intensive training. This is of a great importance for individuals who are not able to perform exercise because of physical handicap or diseases like heart failure.

Importantly, data generated by functional genomics, especially if combined with functional proteomics and the dynamic-dependent studies of the diverse related pathways will not only provide new insight into therapeutic options and research applications but also into clinical implications. Such implications will cover exercise, HF diet, but also other obesity-related factors such as hormones which are worth exploring within the functional genomics context.

## Abbreviations

ECM: extracellular matrix; HF, high-fat; HFHC, HF and high-cholesterol; LF, low-fat; MUP1/Mup1, major urinary protein 1; OXPHOS, oxidative phosphorylation; PGC1α (also known as PPARGC1A), peroxisome proliferator-activated receptor γ coactivator 1 α; *SPARC*/SPARC, secreted protein acidic and rich in cysteine; *Tff2*, trefoil factor 2.
